# Unexpected drug residuals in human milk in Ankara, capital of Turkey

**DOI:** 10.1186/s12884-019-2506-1

**Published:** 2019-10-11

**Authors:** Ayşe Meltem Ergen, Sıddıka Songül Yalçın

**Affiliations:** 1 Department of Family Medicine, Faculty of Medicine, Hacettepe University, Ankara, Turkey; 2 Unit of Social Pediatrics, Department of Pediatrics, Faculty of Medicine, Hacettepe University, Ankara, Turkey

**Keywords:** Breast milk, Drug remnants, Crying, Sleep, Growth, Postnatal maternal weight

## Abstract

**Background:**

Breast milk is a natural and unique nutrient for optimum growth and development of the newborn. The aim of this study was to investigate the presence of unpredictable drug residues in mothers’ milk and the relationship between drug residues and maternal-infant characteristics.

**Methods:**

In a descriptive study, breastfed infants under 3 months of age and their mothers who applied for child health monitoring were enrolled for the study. Information forms were completed for maternal-infant characteristics, breastfeeding problems, crying and sleep characteristics of infants. Maternal and infant anthropometric measurements and maternal milk sample were taken. Edinburgh Postpartum Depression Scale was applied to mothers. RANDOX Infiniplex kit for milk was used for residual analysis.

**Results:**

Overall, 90 volunteer mothers and their breastfed infants were taken into the study and the mean age of the mothers and their infants was 31.5 ± 4.2 years and 57.8 ± 18.1 days, respectively. Anti-inflammatory drug residues in breast milk were detected in 30.0% of mothers and all had tolfenamic acid. Overall, 94.4% had quinolone, 93.3% beta-lactam, 31.1% aminoglycoside and 13.3% polymycin residues. Drugs used during pregnancy or lactation period were not affected by the presence of residues. Edinburgh postpartum depression scores of mothers and crying and sleeping problems of infants were similar in cases with and without drug residues in breast milk. When controlling confounding factors, maternal body mass index alterations were detected to be significantly lower in mothers with anti-inflammatory drug residues in breast milk than in their counterparts (*p* = 0.017).

**Conclusions:**

Our study suggests that there are unpredictable drug residues in the milk of many mothers. Anti-inflammatory drug exposure might affect maternal weight change during the postpartum period. Further studies are required to evaluate the impact of drug residues on maternal and infant health.

## Background

Various pharmaceuticals are given to livestock, fish farms, and beekeeping in order to treat infections, ensure efficiency and growth of products [1]. However, metabolites of veterinary medicinal products given in inappropriate time, dosage and indications, can persist in animals’ muscle, organs and products, and then contaminate people who consume these animals’ products [2]. Remarkably, many drugs’ residuals were reported in food products derived from animal husbandry in different parts of the world [1, 3–9]. The residuals pose a risk of developing some adverse effects including hypersensitivity reaction, antimicrobial resistance, disruption of normal intestinal flora, bone marrow depression and carcinogenicity [10–12]. Therefore, food safety is now universally recognised as a public health priority [13]. These public health concerns are extremely important for mother-foetus pairs during the gestational and lactational periods. Screening all the nutrients consumed by mothers is impossible. However, breast milk can be used for evaluation of the mother-infant environment. Given the possibility of transfer to infants via milk, pharmaceuticals including antibiotics and nonsteroidal anti-inflammatory drugs (NSAID) are carefully prescribed by doctors to mothers during lactation with prescription of doctors [14–16]. Similarly, we predicted that a small amount of drugs might also pass into the breast milk of mothers who consumed contaminated foods [17]. There is only one study about detectable drug residues in the breastmilk of mothers without any history of usage of residual antibiotics in Eskişehir, Turkey [18]. This study supports our hypothesis. However, the status of Ankara, the capital of Turkey, is not known.

Although there are many studies on the effects of exposure to drug residues on human health [19, 20], there is no study in the literature about the impact of drug residuals on maternal anthropometric change after delivery, postpartum depression (PPD) and infant crying and sleeping characteristics. The prevalence of PPD varies from country to country and its incidence varies from 0 to 60% [21]. The prevalence of PPD ranged from 21.2 to 25.0% in Turkey [22]. PPD adversely affects infant mental health [23, 24]. Potential risk factors of PPD include low self-esteem, low social support, low socioeconomic status, stressful life, poor marital relationship, unplanned pregnancy, complications during pregnancy, and prenatal depression [25–27]. However, the pathogenesis of PPD is not fully known [28]. On the other hand, the heptachlor epoxide levels in breast milk samples were found to be positively correlated with Postpartum Bonding Questionnaire, Mother-to-Infant Bonding Scale and indexes of Brief Symptom Inventory indicating some maternal psychopathologies and bonding problems while causing no change in PPD [29]. It is not yet known whether maternal environmental pollutants including pharmaceuticals have an impact on the occurance of PPD.

Sleeping and crying problems among infants were common in the first 3 months postpartum, but the cause could not be elucidated [30]. Maternal drug use can be considered to lead to breast problems and premature discontinuation of breastfeeding and to change the sleeping and crying patterns of infants [10]. Therefore, it can be hypothesised that exposure to veterinarian drugs could affect maternal psychopathologies, breastfeeding success and the well-being of infants.

The primary purpose of the study was to investigate the presence in breastmilk of any residues of drugs that the mother did not take. At the same time it was aimed to investigate the effect of drug residues in breast milk on PPD, breastfeeding success and sleep and crying characteristic of infants.

## Methods

This descriptive study was done in mother-infant pairs who applied for child health follow-up in Hacettepe University İhsan Doğramacı Children’s Hospital between August 2017 and March 2018. Mothers who were breastfeeding their infants and agreed to participate to the study were included. Twin babies and non-breastfed babies were not taken into the study. Any mothers who had taken antibiotics or NSAID for the last 10 days were not included into the study.

This study protocol was examined by Hacettepe University Medical Faculty Non-Interventional Clinical Research Ethics Committee and was approved on August 24, 2017 with the report numbered GO 17/687. Mothers who participated in the study signed voluntary consent form.

### Study protocols

The investigator filled in the mother-infant information form by using a 10-min face-to-face interview technique. The information form contains maternal characteristics [maternal age, education, occupation, history of disease and drug usage (vitamin, antibiotic, anti-parasite, NSAID, vb) during pregnancy and lactation, weight at delivery, smoke exposure] and infant characteristics (age, gender, gestational length, birth type, birth weight, neonatal jaundice presence of concomitant disease, sleep and crying problems according to mothers, feeding type). Edinburgh Postpartum Depression Scale (EPDS) was applied to mothers on admission. Both maternal (weight and height) and infant anthropometric measures (weight, lenght and head circumference) were taken on admission. After breastfeeding for 5 min, approximately 5 mL of human milk were taken into polypropylene tubes by a hand milking method. The collected samples were stored at − 20 °C until analysis.

### Study variables

The history of the baby’s crying was determined by how many hours the baby was crying in the day, whether there was excessive crying or if they had a colic-style crying. “Inconsolable crying” was defined as the mother’s perception of infant crying as excessive and colic-style crying was defined as restlessness, agitation, and crying for at least 2 weeks, 3 days per week, more than 3 h per day and unexplained by another reason.

The body mass index (BMI) of mothers was calculated from values at time of delivery and admission. Anthro program was used to determine “z scores of length, weight and head circumference (respectively, HAZ, WAZ and HCZ respectively) for age and sex” of infants [31].

EPDS was developed by Cox in 1987 [32]. The validity and reliability study of EPDS was conducted by Engindeniz in 1996, in Turkey [33]. The scale consists of 10 items and the items are evaluated as 4-point Likert and scored between 0 and 3. The lowest score is 0 and the highest score is 30. In the evaluation of item 3, 5, 6, 7, 8, 9 and 10, the scoring is reversed.

InfiniPlex biochip kit for milk [Cat no: Ref EV4076, Lot no 9034 (13233EV), RANDOX food diagnostics, London, United Kingdom] was used for residue analysis in milk samples according to manufacturer’s instructions [34]. InfiniPlex test menu is 98% compliant with EU regulated antibiotics and reports 44 assays with 132 contaminants (Additional file 1: Table S1).

### Statistical analysis

Statistical analyses were performed with SPSS 23.0 package program (SPSS Inc., Chicago, IL). Descriptive statistics of cases were given as mean, standard deviation, and distribution rates were given as n, %.

To examine the effect of residues on mother-infant characteristics, the sample was divided into two groups according to the presence of the drug residues in cases having detection rates between 10 and 90%. These drugs are NSAID, aminoglycosides and polymyxin. Chi-square analysis was used to compare independent group ratios. The suitability of the case parameters to normal distribution was examined by Kolmogrov Smirnow test. Differences in mother-infant characteristics were analysed by Student-t test or Mann-Whitney U test according to distribution pattern.

The impact of both the presence of breast milk residue (NSAID, polymyxin AND aminoglycoside) and maternal drug usage (NSAID and antibiotic) during the early postpartum period on the mother’s BMI changes after birth was analysed with general linear models adjusted for maternal age, smoke exposure, gestational length, birth type, maternal BMI at birth, maternal chronic disease, breastfeeding type, infant age.

After controlling for maternal age, maternal chronic disease, smoke exposure, gestational length, birth type, infant’s WAZ score at birth and infant age, general linear models were used to detect both the effect of breast milk residues and maternal self-reported drug use on the infant’s WAZ score on admission in only exclusive breast fed infants.

*P* values below 0.05 were considered statistically significant.

## Results

The study was performed on 90 mothers who were still breastfeeding their babies between 18 and 93 days postpartum.

### General characteristics of mothers

Maternal age, education, working status, smoking status and disease status were given in Table 1. The age range of the mothers was between 24 and 44 years with a mean age of 31.5 years. More than half of the mothers (58.9%) stated no smoking or environmental exposure. Overall, 34.4% of the mothers reported acute or chronic disease; 15 mothers had hypothyroidism, two allergic asthma, two ankylosing spondylitis, and three heart failure. Hypertension was found in 5.6% of mothers, high blood glucose level in 10.1% and urinary tract infection in 13.3% in pregnancy follow-up. 32.2% of the mothers had a disease that required medication. 26.7% of mothers said that they used antibiotics during pregnancy, 32.2% used NSAID and 4.4% steroids. There was a history of use of antibiotics in 42.2% and NSAID in 53.3% of mothers from delivery to the end of the first week. On admission, there was no case taking antibiotics or NSAID within the last 10 days.

**Table 1:** General characteristics of mother-infant pairs, *n* = 90

Maternal characteristics		Infant characteristics	
Age, yrs	31.5 ± 4.2	Gestational duration, weeks	38.2 ± 1.4
Education level, > 12 yrs	63 (70.0)	Birth type, caesarean delivery	62 (68.9)
Occupation, Working	26 (28.9)	Birth order, first child	42 (46.7)
Smoking exposure during lactational period		Infants’ age, days,	57.8 ± 18.1
No smoking	53 (58.9)	Gender, Male	38 (42.2)
Second-hand smoking	28 (31.1)	Intensive care requirement after birth	19 (21.1)
Self smoking	9 (10.0)	Jaundice at the first week, presence	34 (37.8)
Any maternal health problem	31 (34.4)	Infant health problem	18 (20.0)
Drug usage during gestational period			
Vitamin/mineral usage	75 (83.3)		
Antiinflamatuar usage	29 (32.2)		
Antibiotic usage	24 (26.7)		
Any drug usage	86 (95.6)		
Drug usage during lactational period		Feeding type, exclusive breast feeding	72 (80.0)
Vitamin/mineral usage	39 (43.3)	Breastfeeding problems	31 (34.4)
Antiinflamatuar usage^a^	48 (53.3)	Sleeping problems	27 (30.0)
Antibiotic usage^a^	38 (42.2)	Crying duration≥2h	13 (14.4)
Any drug usage	75 (83.3)	Inconsolable crying	7 (7.8)
Edinburg postpartum depression score	7.3 ± 5.3		
Anthropometric values			
Weight gain during pregnancy, kg	15.2 ± 5.4	Birth weight, g	3161 ± 471
Weight at delivery, kg	73.9 ± 11.6	WAZ at birh	−0.30 ± 1.05
Height on admission, cm	163.3 ± 5.7	HCZ on admission	−0.09 ± 1.36
BMI (kg/m^2^) at delivery	27.8 ± 4.5	WAZ on admission	−0.24 ± 1.25
BMI (kg/m^2^) on admission	26.2 ± 4.2	HAZ on admission (*n* = 70)	−0.46 ± 1.52
BMI change from birth to admission	−1.56 ± 3.20	BAZ on admission (n = 70)	−0.02 ± 1.14

Values were mean ± SD or n (%)

^a^only for the first 5–10 days after delivery. On admission, mothers had stated the intake of neither antibiotics nor NSAID for the last 10 days

Overall, 34.4% of mothers stated some breastfeeding problems; cracked nipple (21.1%), insufficient milk (20.0%), breast engorgement (6.7%), breast refusal (7.8%), mastitis (3.3%).

The mean EPDS score of the mothers was 7.3 ± 5.3 points. While 25.6% of the mothers had an EPDS score of 10 points or more, 16.7% had 13 points or more.

### General characteristics of babies

The mean age was 57.8 ± 18.1 days, 38 (42.2%) babies were male and 46.7% were the first child of the family (Table 1). The mean birth weight of the babies was 3161 ± 471 g. Overall, 18 babies (20%) were found to have a disease; eight infants had cardiac pathology (atrial septal defect, ventricular septal defect, patent ductus arteriosus and tetralogy of fallot), and one had hypothyroidism, one had spinal muscular atrophy and one had epilepsy. It was reported that 37.8% of infants had jaundice during the first week of life; however, indirect hyperbilirubinemia levels had been below 12 g/dl. The ratio of exclusively breastfed infants was 80%. Of all, 63 mothers said that their babies were sleeping regularly. The rate of babies crying for 2 h or more per day was 14.4%, and there were only 7 (7.8%) infants who had untimely crying according to the mother’s declaration.

### Drug residues in breast milk samples

No drug residues were detected in only four mothers’ milk.

Some residues of NSAID were found in 30.0% of the mother’s milk (Table 2). All of these cases had tolfenamic acid residues, but in one case 5-OH flunixin, chlormadinone, metamizol and meloxicam residues were detected at the same time.

**Table 2:** Detection rates of drug residues in mother’s milk according to their groups and nomenclature

Residue drug groups	*n* (%)	Positive residue drugs		*Residues, not found in breast milk*
			*n* (%)	
Antiinflammatory	27 (30.0)	5-OH Flunixin	2 (2.2)	*Phenylbutazone*
		Chlormadinone	1 (1.1)	
		Metamizole	1 (1.1)	
		Tolfenamic acid	27 (30.0)	
		Meloxicam	1 (1.1)	
Diaminopirimidine	1 (1.1)	Baquiloprim	1 (1.1)	
		Trimethoprim	1 (1.1)	
Aminoglycoside	28 (31.1)	Spectinomycin	27 (30.0)	*Tobramycin*
		Streptomycin	1 (1.1)	*Apramycin*
		Neomisin	1 (1.1)	*Hygromycin B*
		Gentamisin	1 (1.1)	*Kanamycin*
Quinolone	85 (94.4)	Quinolone^b^	85 (94.4)	
Lincosamide	3 (3.3)	Lincomycin	3 (3.3)	*Pirlimycin*
Sulphonamide	2 (2.2)	Sulphonamides	1 (1.1)	*Sulphapyridine,*
		Sulphamethazine	1 (1.1)	*Sulphaguanidine*
		Dapsone	1 (1.1)	
Macrolide	1 (1.1)	Tylosin	1 (1.1)	*Erythromycin, Spiramycin*
Penicillin	84 (93.3)	Beta-Lactams^a^	84 (93.3)	
Corticosteroid	3 (3.3)	Methylprednisolone	3 (3.3)	*Dexamethasone*
Amphenicol	2 (2.2)	Amphenicol	2 (2.2)	
Aminocoumarin	1 (1.1)	Novobiocin	1 (1.1)	
Antiparasitic	3 (3.3)	Nitroxynil	3 (3.3)	
Polymixin	12 (13.3)	Polymixin	12 (13.3)	
Naphthalene ringed ansamycins	0 (0.0)			*Rifaximin*
Streptogramins	0 (0.0)			*Virginiamycin*
Growth Promoter	0 (0.0)			*Ractopamine*
Polypeptide	0 (0.0)			*Bacitracin*
Tetracycline	0 (0.0)			*Tetracycline*
Cefalosporin	0 (0.0)			*Cefalexin, Cefuroxime*

^a^“Beta-lactams assay” includes “penicillin compounds (ampicillin, amoxicillin, cloxacillin, dicloxacillin, penicillin G, penicillin V and nafcillin) and cephalosporin compounds (cefacetril, cefapirin, cefalonium, cefazolin, ceforperazone, cefquinome and ceftiofur)” with detection limit of 0.35–25.00 ppb

^b^“Quinolones assay” includes “ciprofloxacin, danofloxacin, difloxacin, enrofloxacin, flumequine, marbofloaxacin and oxolinic acid” with detection limit of 12.5–22.5 ppb

Residues of quinolone and beta-lactams (94.4 and 93.3%, respectively) were detected in most of the mothers’ milk (Table 2).

Residues of aminoglycosides were revealed in 28 (31.1%) mothers’ milk samples. Of them 27 had spectinomycin, one had gentamicin. Polymyxin residue was found in 12 (13.3%) breast milk samples.

Three breast milk samples contained lincomycin. Two breast milk samples contained sulphonamide residues; one having dapsone and another having both sulphonamide and sulphamethazine residues. There were two breastmilk samples containing amphenicol residues. One breastmilk sample contained novobiocin. Macrolide residue, tylocin, was revealed in only one mother’s milk. Only one breastmilk sample contained both baquiloprim and trimetoprim in diaminopyridine group drug residue. Nitroxinyl residue, an antiparasitic drug, were detected in three breastmilk samples.

### Maternal-infant characteristics according to the status of NSAID in breastmilk

More than half of mothers had reported the intake of NSAID within the first 5 days after delivery. However, maternal age, educational status, occupation, history of drug use and cigarette contact did not affect the presence of NSAID residue (Table 3). High scores for the EPDS frequency did not differ according to the contact of NSAID. The age, gender, birth type, birth order and gestational length did not differ according to the contact with NSAID.

**Table 3:** Maternal-infant characteristics according to the presence of anti-inflammatory drug residues in breast milk

	Anti-inflammatory drug residues		
Maternal characteristics	Negative (*n* = 63)	Positive (*n* = 27)	*P*
Maternal age, yrs	31.4 ± 4.4	31.5 ± 4.0	0.940
Maternal education> 12 yrs	73.0	63.0	0.340
Working in a revenue-generating business	25.4	37.0	0.264
Anti-inflammatory drug use during breastfeeding period	57.1	44.4	0.268
Antibiotic drug use during breastfeeding period	41.3	44.4	0.780
Vitamin / mineral use during breastfeeding period	41.3	48.1	0.546
Any drug use during breastfeeding period	84.1	81.5	0.764
Maternal cigarette contact, active or passive	38.1	48.1	0.374
Edinburgh postpartum depression score ≥ 13	14.3	22.2	0.368
	7.13 ± 4.98	7.67 ± 5.95	0.658
Mother BMI (kg/m^2^) at delivery	27.9 ± 4.4	27.5 ± 4.8	0.758
Mother BMI (kg/m^2^) on admission	25.8 ± 3.6	27.1 ± 5.2	0.203
Infant characteristics			
Infant age, days	57.6 ± 17.8	58.3 ± 19.1	0.867
Infant age < 60 days	39.7	33.3	0.569
Male sex	41.3	44.4	0.780
Delivery type, cesarean section	71.4	62.9	0.427
Birth order> 1	46.6	48.1	0.854
Gestation time < 38 weeks	27.0	40.7	0.196
Gestation time, weeks	38.2 ± 1.3	38.1 ± 1.5	0.974
Birthweight, WAZ	−0.32 ± 0.85	−0.27 ± 1.41	0.857
Intensive care requirement	17.5	29.6	0.195
Hyperbilirubinemia	31.7	51.9	0.071
Any underlying disease	22.2	14.8	0.421
Sleep disorder	27.0	37.0	0.340
Excessive crying > 2 h	15.9	11.1	0.556
Inconsolable crying	9.5	3.7	0.670
Breastfeeding problem	28.6	48.1	0.073
Exclusive breastfeeding	77.8	85.2	0.421
WAZ score on admission	−0.4 ± 1.2	0.1 ± ±1.4	0.106
HCZ score on admission	−0.2 ± 1.3	0.1 ± 1.4	0.316

Values were mean ± SD or %

While the frequency of neonatal jaundice in the first weeks postpartum was 51.9% in infants with NSAID residues in breast milk, it was 31.7% in the no residue detected group (*p* = 0.071). Detection of NSAID residues slightly increased breastfeeding problems (*p* = 0.073). The crying and sleep characteristics of the infants were not influenced by the presence of NSAID residue in milk (Table 3).

### Maternal-infant characteristics according to the presence of antibiotic residues in breast milk

Nearly half of mothers had reported the intake of antibiotics after delivery within the first 5 days. However, no mother had stated the use of quinolone, aminoglycoside or polymyxin for the previous 1 year. Detection rates of aminoglycoside and polymyxin antibiotic residues were between 10 and 90% and further evaluation was done.

Maternal age, educational status, working status, history of any drug use during lactation, smoke exposure and EPDS≥13 were similar in groups with or without containing aminoglycoside antibiotic residues in breast milk (Table 4). Similarly, the presence or absence of polymyxin drug residue in breast milk did not differ according to maternal and infant baseline characteristics. Neither the detection of aminoglycoside nor polymyxin drug residues in breast milk affect the need for intensive care for the baby, the history of jaundice, breastfeeding problems, sleep and crying problems (Table 4).

**Table 4:** Maternal-infant characteristics according to the presence of aminoglycoside drug residues in breast milk

	Aminoglycoside residues			Polymixin residues		
Maternal characteristics	Negative (*n* = 62)	Positive (*n* = 28)	*P*	Negative (*n* = 78)	Positive (*n* = 12)	*p*
Age, yrs	32.0 ± 4.4	30.5 ± 3.6	0.132	31.7 ± 4.3	30.2 ± 3.9	0.255
Education> 12 yrs	69.3	71.2	0.842	71.8	58.3	0.335
Working mothers	30.6	25.0	0.584	29.5	25.0	1.000
NSAID use during postpartum period	59.7	39.3	0.073	56.4	33.3	0.136
Antibiotic drug use during postpartum period	41.9	42.9	0.935	41.0	50.0	0.558
Vitamin/mineral use during postpartum period	40.3	50.0	0.391	41.0	58.3	0.260
Any drug use during postpartum period	82.3	85.7	0.769	83.3	83.3	1.000
Maternal cigarette exposure, active or passive	38.7	46.4	0.491	42.3	33.3	0.755
EPDS on admission ≥13	14.5	21.4	0.542	17.9	8.3	0.682
EPDS on admission	6.87 ± 4.65	8.21 ± 6.42	0.265	7.60 ± 5.33	5.25 ± 4.49	0.151
Maternal BMI at birth, kg/m^2^	28.0 ± 4.8	27.2 ± 3.8	0.420	27.8 ± 4.6	27.7 ± 3.7	0.945
Maternal BMI at visit, kg/m^2^	26.2 ± 4.4	26.2 ± 3.7	0.993	26.3 ± 4.3	25.8 ± 3.5	0.739
Infant characteristics						
Age, day	59.0 ± 18.1	55.1 ± 18.2	0.346	58.1 ± 18.4	56.0 ± 16.8	0.706
Infant age < 60 days	33.9	46.4	0.255	37.2	41.7	0.765
Male sex	40.3	46.4	0.587	41.0	50.0	0.558
Delivery, Cesarean section	72.6	60.7	0.260	67.9	75.0	0.747
Birth order> 1	48.4	42.9	0.626	48.7	33.3	0.320
Gestation lenght < 38 weeks	33.9	25.0	0.400	32.1	25.0	0.747
Gestational lenght, weeks	38.0 ± 1.3	38.6 ± 1.5	0.056*	38.1 ± 1.4	38.6 ± 1.2	0.254
Birthweight, WAZ	−0.35 ± 1.06	−0.19 ± 1.04	0.494	−0.32 ± 10.2	−0.23 ± 1.26	0.794
Intensive care requirement	17.7	28.6	0.244	19.2	33.3	0.271
Neonatal hyperbilirubinemia	38.7	35.7	0.786	38.5	33.3	1.000
Any underlying disease	16.1	28.6	0.170	35.9	25.0	0.534
Sleep disorder	30.6	28.6	0.842	29.5	33.3	0.747
Excessive crying > 2 h	14.5	14.3	0.977	15.4	8.3	1.000
Inconsolable crying	9.7	3.6	0.428	8.9	0.0	0.587
Breastfeeding problem	32.3	39.3	0.516	33.3	41.6	0.745
Exclusive breastfeeding	77.4	85.7	0.362	79.5	83.3	1.000
WAZ score on admission	−0.3 ± 1.3	− 0.2 ± 1.2	0.916	− 0.2 ± 1.3	− 0.3 ± 1.0	0.914
HCZ score on admission	0.02 ± 1.3	−0.3 ± 1.4	0.251	0.01 ± 1.4	−0.7 ± 1.2	0.092

Values were mean ± SD or %

### Veterinary drug residuals and anthropometric evaluations of mother-infant pairs

The BMI of the mother at delivery and on admission did not differ according to the presence of the residues of NSAID, aminoglycoside and polymyxin in her milk (Table 3). However, in multivariate analysis, compared to counterparts, maternal BMI change from birth to delivery was observed significantly lower both in cases with NSAID residuals (− 0.51 ± 0.53, − 2.07 ± 0.35 kg/m^2^ respectively; *p* = 0.017) and in mothers with history of NSAID intake (− 0.57 ± 0.47, − 2.01 ± 0.46 kg/m^2^ respectively; *p* = 0.038) after adjusting confounding factors (Table 5). Similarly, compared to unexposed groups, maternal history of self-use of any antibiotics after delivery decreased maternal BMI improvement after delivery after adjusting control groups (Table 6).

**Table 5:** Impact of “breast milk NSAID residues” and “maternal NSAID usage during early postpartum period” on the on the maternal BMI change after birth and infant WAZ and HCZ values on admission, multivariate analysis

Breast milk residue	Maternal NSAID usage during postpartum period			Overall	P for residue	P for maternal self-use	Interaction between residue and self-use
		Absence	Presence				
Maternal BMI change after birth^a^							
NSAID	–	−2.77 ± 0.55	−1.38 ± 0.46	− 2.07 ± 0.35	0.017	0.038	0.938
NSAID	+	−1.26 ± 0.73	0.23 ± 0.81	− 0.51 ± 0.53			
Overall		−2.01 ± 0.46	−0.57 ± 0.47				
Infant’s WAZ^b^							
NSAID	–	−0.13 ± 0.20	−0.10 ± 0.16	− 0.11 ± 0.13	0.555	0.895	0.805
NSAID	+	0.07 ± 0.23	−0.02 ± 0.32	0.03 ± 0.19			
Overall		−0.03 ± 0.15	−0.06 ± 0.18				

Values were mean ± standart error of mean

^a^ Analysed with general linear models adjusted for maternal age, smoke exposure, gestational length, birth type, maternal BMI at birth, maternal disease, breastfeeding type, infant age

^b^ Exclusive breast fed infants were enrolled and analysed with general linear models adjusted for maternal age, maternal disease, smoke exposure, gestational length, birth type, infant’s WAZ at birth, infant age

**Table 6:** Impact of “breast milk antibiotic residues” and “any history of maternal antibiotic usage during early postpartum period” on the maternal BMI change after birth and infant WAZ and HCZ values on admission, multivariate analysis

Breast milk residue	Maternal antibiotic usage during postpartum period			Overall	P for residue	P for maternal self-use	Interaction between residue and self-use
		Absence	Presence				
Maternal BMI change after birth^a^							
Polymyxin	–	−2.53 ± 0.40	−0.15 ± 0.48	−1.34 ± 0.30	0.764	0.004	0.962
Polymyxin	+	−2.81 ± 1.08	−0.36 ± 1.09	−1.59 ± 0.77			
Overall		−2.67 ± 0.57	−0.26 ± 0.59				
Infant’s WAZ^b^							
Polymyxin	–	−0.20 ± 0.15	0.18 ± 0.17	−0.01 ± 0.11	0.525	0.176	0.920
Polymyxin	+	−0.42 ± 0.39	0.02 ± 0.39	−0.20 ± 0.27			
Overall		−0.31 ± 0.21	0.10 ± 0.21				
Maternal BMI change (kg/m^2^) after birth^a^							
Aminoglycoside	–	−2.72 ± 0.45	−0.39 ± 0.53	−1.55 ± 0.34	0.364	< 0.001	0.889
Aminoglycoside	+	−2.23 ± 0.67	0.27 ± 0.79	−0.98 ± 0.52			
Overall		−2.47 ± 0.40	−0.06 ± 0.47				
Infant’s WAZ^b^							
Aminoglycoside	–	−0.17 ± 0.16	0.17 ± 0.20	0.00 ± 0.13	0.585	0.067	0.751
Aminoglycoside	+	−0.36 ± 0.25	0.12 ± 0.25	−0.12 ± 0.18			
Overall		−0.26 ± 0.15	0.15 ± 0.16				

Values were mean ± standart error of mean

^a^ Analysed with general linear models adjusted for maternal age, smoke exposure, gestational length, birth type, maternal BMI at birth, maternal disease, breastfeeding type, infant age

^b^ Exclusive breast fed infants were enrolled and analysed with general linear models adjusted for maternal age, maternal disease, smoke exposure, gestational length, birth type, infant’s WAZ at birth, infant age

There were no differences in WAZ of infant at birth and on admission according to presence of selected veterinary drug residues (Table 4). Similarly, no differences were detected in WAZ scores of infants according to NSAID exposure in multivariate analysis (Table 5). When confounding factors were controlled, WAZ scores of infants with antibiotic residues in their breast milk were similar to unexposed groups (Table 6).

## Discussion

In our study, although it was not used by the mothers themselves, nearly all mother’s milk samples contained some residuals from medicines used in the area of livestock ​​fattening. Only a minority of mothers milk (*n* = 4) had no drug residues. This is the second article showing the residues of veterinary drugs in human milk [18].

Remnants of NSAID were found in the milk of 27 of 90 mothers included in our study in Ankara, all containing tolfenamic acid. Tolfenamic acid is for the management of both acute and chronic pain in animals and indicated a long half-life in sheep [35]. Previous studies didn’t detect any remnants of tolfenamic acid in 83 studied human milk samples in Eskişehir [18]. However, one or two of samples contained chlormadione, metamizole and meloxicam in both the Ankara and Eskişehir studies.

Most of the drug residues in our study in Ankara were found to be quinolone and beta-lactam antibiotics in our study in Ankara. The Eskişehir study revealed 85.5% of breast milk with beta-lactam residues, however, a lower detection rate for quinolone residues (14.5%) [18]. Of the human’s milk in our study, 31.1% aminoglycoside residues and 3.3% polymyxin residues were found and no residues in the assays of tetracycline, polypeptide and growth promotor drugs were detected in our study. The Eskişehir study revealed 4.8% polymyxin, and only 2.4% aminoglycoside (tobramycin) [18]. This might be explained by different contamination of consumed foods in different cities. A minority of samples had amphenicol, sulphonamide and corticosteroid residues in both cities.

To explicate veterinary drug residues in human milk, we must examine studies in animal products. Honey samples from 22 different regions of Turkey such as Muş, Bingöl, Şemdinli, Yüksekova and Marmaris-Muğla were investigated and although the use in beekeeping was not legal, tetracycline, streptomycin and sulfonamide group antibiotics were detected in 15% of the samples [36, 37]. One study analysed 240 pasteurized and raw milk samples sold in an Ankara market and detected oxytetracycline, penicillin G and neomycin in one milk sample above the maximum residual levels (MRL) allowed [38]. However, another study in Ankara in 2018 revealed 52.5% of milk samples had beta-lactam antibiotic residues above the detection limit (0.1 μg/kg) and 8.75% above 3 μg/kg [39]. However, in another study conducted in Ankara in 2010, 127 chicken meat and 104 beef samples from three different regions were selected randomly and quinolone group antibiotic residues were detected in 45.7% of chicken meat samples and 57.7% of beef meat samples [7]. This may explain the detection of quinolone and beta-lactam in human milk in our study. Residues of veterinary medicinal products are a global problem [40]. In some African countries veterinary drug residues can be as high as 94% in foods of animal origin. In 10 provinces of China, 0.5, 47.2 and 20.1% of 199 raw milk samples were reported to be positive for beta-lactams, quinolones and sulfonamides, respectively at the detectable level [41]. A study from Nepal detected 81% samples positive for amoxicillin, 41% for sulfadimethoxine, 27% for penicillin G, and 12% for ampicillin in 140 fresh milk [6]. These reports can be explained by unnecessary use of antibiotics, ignorance of the prescribed drug use and the recommended pre-slaughter time by the people who practice fattening during the last decade [7, 42].

In our study, no significant relationship was found between the presence of drug residues in mothers’ milk and PPD. In addition, antibiotic and NSAID residues found in breast milk did not have any influence on crying and sleep problems. However, in cases with NSAID residues, neonatal jaundice and breastfeeding problems were detected slightly higher (*p* = 0.071 and *p* = 0.073) which was not found to be statistically significant. NSAIDs are consumed widely during the early lactational period [17]. On the other hand, it is known that NSAIDs have a risk for renal injury, constriction of the ductus arteriosus, necrotizing enterocolitis and intracranial haemorrhage when used in the third trimester of gestation [11]. Therefore, further detailed studies in lactating mothers with pharmaceutical intake are necessary to clarify this hypothesis.

It is observed that the presence of NSAID residues has an impact on postnatal maternal weight regulation and may slow down the return of the mother to pre-pregnancy weight. In addition, in the present study, maternal self-use of antibiotics was shown to have a similar effect on maternal postpartum weight change. There has been no study evaluating the influence of analgesic drug usage on the mother’s BMI. However, recent studies showed the association of antibiotic use with obesity in childhood period [43, 44]. Rogawski et al. reported that antibiotic use in the first 6 months of life was associated with increased weight, especially among children with two or more exposures to macrolides, metronidazole, and cephalosporins [43]. Similarly, Block et al. found that antibiotic use at < 24 months of age was associated with a slightly higher body weight at 5 years of age [44]. The changes in anthropometric parameters are explained by the influence of antibiotics on the diversity and composition of the gut microbiota, which can persist long after the treatment and can modify metabolism and energy harvest of the diet [45–47]. Similarly, antibiotics including tetracyclines, macrolides, avoparcin and penicillins have been commonly used in livestock to promote growth and increase weight gain [48]. We detected no influence of antibiotic residues on infant’s antropometric evaluations. This might be due to the small amount of residues in breast milk. However, a cause-effect relationship could not be established between drug residues and the health parameters of the mother and child with a single assessment in our study. Because of infant and maternal health concern, more extensive studies are needed to illuminate this outcome. Nonetheless, this predicted impact should be particularly emphasised and unnecessary drug use should be prevented.

### Strengths and limitations

Our study is the first comprehensive one examining the presence of veterinarian drug residues in human milk and the relationship between the baby and maternal problems. We enrolled nursing mothers with babies younger than 3 months and receiving no complementary food. This way, the infants who might be exposed to different pollutants through food were not factored into the study. Our study also showed drug residues similar to contaminant in animal husbandry in the experimental region. Given that one milk sample from nursing mothers shows the current environmental status of mother-infant pairs, however, as a limitation we could not determine the past contamination.

Significant time-dependent changes in water, lipid and protein content of human milk are well known. Therefore, carry-over content though milk might be change in foremilk and hindmilk [49]. We planned to see content of foremilk in the natural situation. Nursing a baby stimulates the maternal oxytocin response and provides milking by a physiological mechanism. Considering this fact, we took breastmilk at the fifth minute of sucking by a hand milking method. So, we examined only the presence of contaminants in foremilk. These same techniques and methodologic procedures could be used in further studies. This will enable method standardisation.

In our study, the amount of drug residues was not determined and this was a limitation of the study. Previous studies had evaluated assay methods to determine drugs residues in animal products following self-consumption [50]. However, there is no kit specific to human milk. Therefore, we used InfiniPlex Biochip kit intended to test milk meant to be cconsumed as food [34]. Detection of very low doses of residuals with traceable technology is the strength of our study (Additional file 1: Table S1). Multiresidue methods can make visible the problems in different areas [51]. Our descriptive study is performed for the evaluation of problems and screened for multiple drug residues. Our study points to the possibility for further studies on this subject. Further quantitative studies can be planned on these detected residues.

## Conclusions

The majority of the mothers included in the study had drug residues in their breast milk. These results show that mothers are exposed to drugs in an unexpected manner through foods and what’s more, some of these drugs detected in breast milk are prohibited in humans. In our study, the impact of residues in the mother’s milk on baby sleep and crying problems, breastfeeding problems and PPD were not found to be significant. However, drug residues were found to influence the regulation of the anthropometric parameters of the mother.

Preventing unnecessary drug exposure in mother-baby pairs is extremely important for healthy generations. Therefore, veterinary medicinal products must be used in appropriate indications; obedience to appropriate waiting time must be monitored.

## Supplementary information

**Additional file 1: Table S1.** Detection limits of residues for Randox biochip array technology.

## Data Availability

The datasets used during the current study are available from the corresponding author on reasonable request.

## Abbreviations

AB: Antibiotic
BMI: Body mass index
EPDS: Edinburgh Postpartum Depression Scale
EU: European Union
HAZ: Z scores of length
HCZ: Z scores of head circumference
NSAID: Nonsteroid antiinflammatory drugs
PP: Postpartum
PPD: Postpartum depression
SD: Standard deviation
SPSS: Statistical Package for the Social Sciences
WAZ: Z scores of weight
