# Correction to: Unexpected drug residuals in human milk in Ankara, capital of Turkey

**DOI:** 10.1186/s12884-020-03063-y

**Published:** 2020-07-06

**Authors:** Ayşe Meltem Ergen, Sıddıka Songül Yalçın

**Affiliations:** 1 Department of Family Medicine, Faculty of Medicine, Hacettepe University, Ankara, Turkey; 2 Unit of Social Pediatrics, Department of Pediatrics, Faculty of Medicine, Hacettepe University, Ankara, Turkey

**Correction to: BMC Pregnancy Childbirth 19, 348 (2019)**

**https://doi.org/10.1186/s12884-019-2506-1**

Following publication of the original article [1], the authors would like to change the Reference 34.

In this Correction, correct and incorrect versions are shown.

Incorrect: Jin X, Tian X, Liu Z, Hu H, Li X, Deng Y, Li N, Zhu J: Maternal exposure to arsenic and cadmium and the risk of congenital heart defects in offspring. Reprod Toxicol 2016, 59:109–116.

Correct: RANDOX food diagnostics. Biochip Array Technology. Available from: https://www.randoxfood.com/biochip/. [Cited 2019 May 11]
