# Supplementary material for: Unexpected drug residuals in human milk in Ankara, capital of Turkey
Source: BMC Pregnancy Childbirth. 2019 Oct 11;19:348. doi: 10.1186/s12884-019-2506-1 (PMC6788077; doi:10.1186/s12884-019-2506-1)
Supplement: Supplementary file 1 — Additional file 1: Table S1. Detection limits of residues for Randox biochip array technology. [file 12884_2019_2506_MOESM1_ESM.docx]

**Additional file 1. Detection limits of residues for Randox biochip array technology**

| Assay | Compound | Decision Level (ppb) | EU Regulated |
| --- | --- | --- | --- |
| Quinolones | Enrofloxacin | 12.50 | Y |
|  | Ciprofloxacin | 13.80 | Y |
|  | Danofloxacin | 10 | Y |
|  | Difloxacin | 19 | Y |
|  | Marbofloxacin | 9 | Y |
|  | Oxolinic Acid | 12 | Y |
|  | Flumequine | 21 | Y |
| Beta-Lactams | Benzylpenicillin | 1 | Y |
|  | Ampicillin | 2 | Y |
|  | Dicloxacillin | 1 | Y |
|  | Amoxicillin | 1.3 | Y |
|  | Cloxacillin | 2.5 | Y |
|  | Oxacillin | 1.3 | Y |
|  | Nafcillin | 6.5 | Y |
|  | Penicillin V | 0.5 | N/A |
|  | Cephalonium | 0.35 | Y |
|  | Cefoperazone | 1.6 | Y |
|  | Cephapirin | 2.4 | Y |
|  | Cefquinome | 11.6 | Y |
|  | Ceftiofur | 29.2 | Y |
|  | Cefacetril | 16 | Y |
|  | Cefazolin | 22 | Y |
| Cephalexin | Cephalexin | 13.2 | Y |
| Erythromycin | Erythromycin | 1.5 | Y |
|  | Gamithromycin | 8 | Y |
|  | Tulathromycin | 40 | Y |
|  | Oleandomycin | 14 | N/A |
| Spiramycin | Spiramycin | 1.7 | Y |
|  | Neospiramycin | 6 | Y |
| Tylosin | Tylosin A | 32 | Y |
|  | Tilmicosin | 34 | Y |
| Lincomycin | Lincomycin | 4.3 | Y |
| Pirlimycin | Pirlimycin | 5 | Y |
| Neomycin | Neomycin | 11 | Y |
|  | Paromomycin | 2.3 | Y |
| Streptomycin | Streptomycin | 23 | Y |
|  | Dihydrostreptomycin | 14 | Y |
| Gentamycin | Gentamicin | 11 | Y |
|  | Gentamycin C1 | 4.8 | Y |
|  | Gentamycin C1a | 11 | Y |
|  | Gentamycin C2 | 6 | Y |
| Kanamycin | Kanamycin A | 4 | Y |
| Spectinomycin | Spectinomycin | 3 | Y |
| Amphenicols | Chloramphenicol | 0.22 | Y |
|  | Thiamphenicol | 1.6 | Y |
|  | Florfenicol | 0.4 | Y |
| Trimethoprim | Trimethoprim | 7.9 | Y |
| Baquiloprim | Baquiloprim | 7.7 | Y |
| Rifaximin | Rifaximin | 1 | Y |
| Apramycin | Apramycin | 4.4 | Y |
| Virginiamycin M1 | Virginiamycin M1 | 1 | Y |
| Tobramycin | Tobramycin | 4 | Y |
| Tetracyclines | Tetracycline | 13 | Y |
|  | Chlortetracycline | 10 | Y |
|  | Oxytetracycline | 7 | Y |
|  | Doxycycline | 22.5 | Y |
| Polymixins | Colistin | 1 | Y |
|  | Polymixin B | 0.15 | Y |
| Bacitracin | Bacitracin | 1.4 | Y |
| Cefuroxime | Cefuroxime | 7 | N/A |
| 5-hydroxy Flunixin | 5-OH Flunixin | 0.5 | Y |
|  | Flunixin | 0.7 | N/A |
| Meloxicam | Meloxicam | 8.3 | Y |
| Metamizole | Metamizole (Dipyrone) | 7.4 | Y |
| Tolfenamic Acid | Tolfenamic Acid | 27 | Y |
| Phenylbutazone | Phenylbutazone | 1 | Y |
|  | Oxyphenbutazone | 0.8 | N/A |
| Chlormadinone | Chlormadinone | 0.3 | Y |
| Methylprednisolone | Methylprednisolone | 0.4 | Y |
|  | Prednisolone | 2 | N/A |
| Sulfaguanidine | Sulfaguanidine | 7 | Y |
| Sulphapyridine | Sulphapyridine | 1.2 | Y |
|  | Sulfaethoxypyridazine | 16 | Y |
|  | Sulfamethoxypyridazine | 7.7 | Y |
|  | Sulfamoxol | 22 | Y |
|  | Sulfasalazine | 1 | Y |
|  | Sulfanitran | 53 | Y |
|  | Sulfathiazole | 33 | Y |
|  | Sulfamonomethoxine | 82 | Y |
| Sulfamethazine | Sulfamethazine | 0.7 | Y |
|  | Sulfamerazine | 14 | Y |
| Sulphonamides | Sulphathiazole | 10 | Y |
|  | Sulfaquinoxaline | 6.4 | Y |
|  | Sulfadimethoxine | 3 | Y |
|  | Sulfacetamide | 3.7 | Y |
|  | Sulphadoxine | 2 | Y |
|  | Sulfabenzamide | 0.6 | Y |
|  | Sulfamethoxazole | 2.1 | Y |
|  | Sulfamonomethoxine | 1.5 | Y |
|  | Sulfachlorpyridazine | 3.8 | Y |
|  | Sulfadiazine | 9 | Y |
|  | Sulfamethoxypyridazine | 35 | Y |
|  | Sulfisoxazole | 1.5 | Y |
|  | Sulfamerazine | 21 | Y |
|  | Sulfamethizole | 11.5 | Y |
|  | Sulfameter | 4 | Y |
|  | Sulfamoxol | 34 | Y |
|  | Sulfanitran | 5.1 | Y |
|  | Sulfaphenazole | 2.6 | Y |
|  | Sulfatroxazole | 2 | Y |
|  | Sulfisomidine | 11 | Y |
| Dapsone | Dapsone | 0.9 | Y |
| Nitroxynil | Nitroxynil | 0.4 | Y |
| Aflatoxin M1 | Aflatoxin M1 | 0.03 | Y |
| Novobiocin | Novobiocin | 4.2 | Y |
| Ractopamine | Ractopamine | 0.3 | Y |
| Dexamethasone | Dexamethasone | 0.2 | Y |
|  | Betamethasone | 0.7 | Y |
| Hygromycin B | Hygromycin B | 52 | Y |
| Melamine | Melamine | 373 | Y |
